# Multisite learning of high-dimensional heterogeneous data with applications to opioid use disorder study of 15,000 patients across 5 clinical sites

**DOI:** 10.1038/s41598-022-14029-9

**Published:** 2022-06-30

**Authors:** Xiaokang Liu, Rui Duan, Chongliang Luo, Alexis Ogdie, Jason H. Moore, Henry R. Kranzler, Jiang Bian, Yong Chen

**Affiliations:** 1 Department of Biostatistics, Epidemiology and Informatics, University of Pennsylvania Perelman School of Medicine, 423 Guardian Drive, Philadelphia, PA 19104 USA; 2 Department of Biostatistics, Harvard T.H. Chan School of Public Health, Harvard University, Boston, MA USA; 3 Division of Public Health Sciences, Washington University School of Medicine in St. Louis, St. Louis, MO USA; 4 Department of Medicine, Department of Biostatistics, Epidemiology and Informatics, University of Pennsylvania Perelman School of Medicine, Philadelphia, PA USA; 5 Department of Computational Biomedicine, Cedars-Sinai Medical Center, Los Angeles, CA 90096 USA; 6 Department of Psychiatry, University of Pennsylvania Perelman School of Medicine and the VISN 4 MIRECC, Crescenz VAMC, Philadelphia, PA USA; 7 Department of Health Outcomes and Biomedical Informatics, University of Florida Health Cancer Center, Gainesville, FL USA

**Keywords:** Epidemiology, Outcomes research

## Abstract

Integrating data across institutions can improve learning efficiency. To integrate data efficiently while protecting privacy, we propose **A** one-shot, summary-statistics-based, **D**istributed **A**lgorithm for fitting **P**enalized (ADAP) regression models across multiple datasets. ADAP utilizes patient-level data from a lead site and incorporates the first-order (ADAP1) and second-order gradients (ADAP2) of the objective function from collaborating sites to construct a surrogate objective function at the lead site, where model fitting is then completed with proper regularizations applied. We evaluate the performance of the proposed method using both simulation and a real-world application to study risk factors for opioid use disorder (OUD) using 15,000 patient data from the OneFlorida Clinical Research Consortium. Our results show that ADAP performs nearly the same as the pooled estimator but achieves higher estimation accuracy and better variable selection than the local and average estimators. Moreover, ADAP2 successfully handles heterogeneity in covariate distributions.

## Introduction

Electronic health records (EHR), which routinely incorporate information from health care providers and medical devices^1^, contain information about patients’ diagnoses, laboratory test results and medication use and are important resources for biomedical and clinical research^2–4^. With the wide adoption of EHR systems throughout the United States and other countries, there is a growing need to integrate data horizontally from different institutions, i.e., combining data with the same set of features but different patient populations^5^. Such integration can greatly enrich the study population, increase statistical power, reduce the potential for regional bias, and provide opportunities to study rare medical conditions.

However, data integration across institutions has many practical challenges. First, collaborating institutions must adopt data harmonization procedures to facilitate a commonly applicable data analysis approach. There have been many efforts in large research networks to develop common data models (CDM) that create a unified data structure and variable definitions for all the collaborating institutions. For example, the Observational Medical Outcomes Partnership (OMOP) CDM^6,7^ was developed by the Observational Health Data Sciences and Informatics (OHDSI) network to standardize patient records in a consistent format.

Due to privacy protections, it is often not feasible to share individual-level patient data across multiple sites. Distributed algorithms (also known as federated learning^8^ algorithms) that coordinately execute a computing task at each site can bypass the need of sharing individual-level data to achieve data integration by sharing only summary-level statistics. Some recent, exciting developments in the related areas of statistics and machine learning provide the potential for models that incorporate data across multiple datasets in a distributed manner. For example, Chen et al.^9^ developed a distributed algorithm for general linear regression and Luo et al.^10^ proposed a distributed linear mixed model. Some of the distributed algorithms require iterative communication across datasets until they converge, e.g., GLORE^11^ for logistic regression and WebDISCO^12^ for a Cox proportional hazards model. However, multiple rounds of communication can impose a high communication cost, which is measured by the total number of digits transferred and the time and labor cost spent in communications, to complete the task and requires cooperation among institutions, which may not be feasible for some applications. Thus, one-shot methods that require only a single round of communication are preferred. Among a variety of recently developed one-shot algorithms, a commonly used approach is to average all of the local estimates (e.g., Zhang et al.^13^, Lee et al.^14^, Battey et al.^15^, Dobriban and Sheng^16,17^). The potential limitation of the averaging-type of methods is their lack of accuracy when studying rare conditions. For example, in^18^ the authors demonstrated that when the event rate is low the averaging-type estimator can lead to non-negligible bias and large variance. Another approach constructs a surrogate of the global likelihood function using the individual-level data in a lead site and summary-level statistics from the collaborating sites (e.g., Jordan et al.^19^, Wang et al.^20^, Duan et al.^21^, and Duan et al.^22^). In addition, efficient one-shot distributed algorithms have been proposed to deal with zero-inflated count data^23^ and time-to-event data^18^.

Most of the aforementioned work involves low-dimensional regression models where the number of predictors is smaller than the sample size. A unique challenge in the era of big data is high dimensionality, where the number of parameters can be extremely large and much bigger than the sample size. For example, in genomic studies thousands of genetic variants are observed for each subject, and methods are needed to select the truly influential variables. Penalized regression is one of the most commonly used techniques for variable selection. It maximizes the goodness of fit of the model while controlling the model complexity by restricting the regression coefficients. For example, lasso regression^24^ allows simultaneous model estimation and variable selection by adding an *l*_1_ penalty of the regression coefficients. Ridge regression^25^ exploits an *l*_2_ penalty to handle the high collinearity among covariates and elastic-net^26^ flexibly combines the *l*_1_ and *l*_2_ penalties so that strongly correlated covariates are included in or excluded from the model together. Some distributed algorithms are proposed for penalized regressions (e.g., Lee et al.^14^, Battey et al.^15^, Dobriban and Sheng^17^, and Fan et al.^27^). However, these methods either assume a homogeneous application scenario that can introduce bias into the estimation in the presence of heterogeneity across sites^28,29^ or cannot accommodate rare outcomes, underscoring the need for a framework that deals with the two issues simultaneously.

In this article, we propose a distributed algorithm for penalized regression. Different from the existing methods^19,20,27^, our method accounts for heterogeneity in covariate distributions across multiple sites by incorporating the second-order gradient information when creating the surrogate objective function. To be compatible with the structure of the surrogate function, a modified cross-validation strategy is used to tune the level of regularization. We evaluate the performance of the proposed method using both simulation and a real-world application to study risk factors for opioid use disorder (OUD) with data from five participating sites of the OneFlorida Clinical Research Consortium^30^, a clinical data research network contributing to the national Patient-Centered Clinical Research Network (PCORnet)^31^. We chose to focus on OUD because of the substantial implications the disorder has for public health. Between 1990 and 2010, U.S. opioid analgesic prescriptions increased by a factor of 10^32^, contributing to an epidemic of opioid misuse, abuse, and overdose deaths^32–34^. By 2018, 3.7% of U.S. adults reported past-year misuse of a prescription opioid pain reliever^35^. With the increase in misuse of opioids, the prevalence of prescription OUD among U.S. adults reached 2.1 million (or 0.9%)^36^. During the COVID-19 pandemic, despite decreases in emergency department visits for other medical emergencies, during 2020 the rates of opioid overdose-related visits in six healthcare systems increased^37^. These findings are consistent with a widespread increase in opioid-related complications during the pandemic.

The OneFlorida data repository integrates multiple data sources from its participating healthcare organizations and provides real-world data to support biomedical and clinical research^38–40^. For this study, we extracted EHRs (covering patient records from 01/01/2012 up to 07/31/2020) from the 5 participating sites for 15,000 patients who had chronic pain and an opioid prescription (including buprenorphine, codeine, fentanyl, hydromorphone, meperidine, methadone, morphine, oxycodone, tramadol, and hydrocodone) and no cancer or OUD diagnosis before their first opioid prescription. Among these patients who were exposed to an opioid, we define a case of OUD as having a first diagnosis of OUD after their first prescription and define a control as having no diagnosis of OUD during the entire time window. A list of risk factors was compiled from the literature and extracted from the database, including basic demographic features such as age, gender and race, and co-occurring diagnoses, e.g., depression and sleep disorder (see Supplementary Table 2 for all 42 covariates). A logistic lasso regression is then applied to locate truly influential risk factors. These numerical study results show that, by adding the second-order gradient information, estimation, prediction, and variable selection of ADAP2 are improved compared to some alternative methods and ADAP2 is robust to the heterogeneity in covariate distributions.

## Results

### Overview of the ADAP method

The ADAP method aims to efficiently learn a global parsimonious association relationship between an outcome of interest and a large amount of risk factors through integrate data across multiple sites while protecting privacy. Briefly, we model the association using a regression model with coefficient $\beta$ and a loss function (objective function) is used to measure the goodness of fit of the model. ADAP utilizes patient-level data from a lead site and incorporates the first-order (ADAP1) and second-order gradients (ADAP2) of the objective function from collaborating sites to construct a surrogate objective function^19,20^ at the lead site. Then, after applying proper regularizations to the surrogate objective function to select truly influential risk factors, we obtain the estimates of the association coefficients $\beta$ by optimizing the penalized surrogate objective function.

To demonstrate the property of ADAP, we compared ADAP1 $\hat{\beta }^{( 1 )}$ and ADAP2 $\hat{\beta }^{( 2 )}$ to several benchmark methods, including the local estimator $\hat{\beta }_{1}$ obtained from a single dataset (i.e., the lead site dataset), the pooled estimator $\hat{\beta }_{N}$ obtained from the combined patient-level data across all sites, and the average estimator $\hat{\beta }_{ave}$ obtained by averaging all local estimators. The pooled estimator is considered as a gold standard as it directly uses all the patient-data without constrains on data-sharing, but it is not available in practice. The details of ADAP, as well as the benchmark methods can be found in “Methods”. For illustration, in the following we mainly consider the lasso logistic regression.

### Evaluation of estimation accuracy and variable selection through simulation studies

We evaluate the performance of ADAP under various settings to see the effects of number of sites *K* (Setting 1), heterogeneous covariate distribution (Setting 2), lead site’s sample size *n*_1_ (Setting 3), and a shared sample size *n* by each site (Setting 4) on the performance of the method. Except for the estimation performance, which is measured by the Euclidean distance of the estimate to its true value, we also tested the variable selection ability in Setting 5 with multiple levels of association magnitude by calculating the true positive rate and false positive rate. In all five settings, we let site 1 be the lead site.

**Figure 1:**
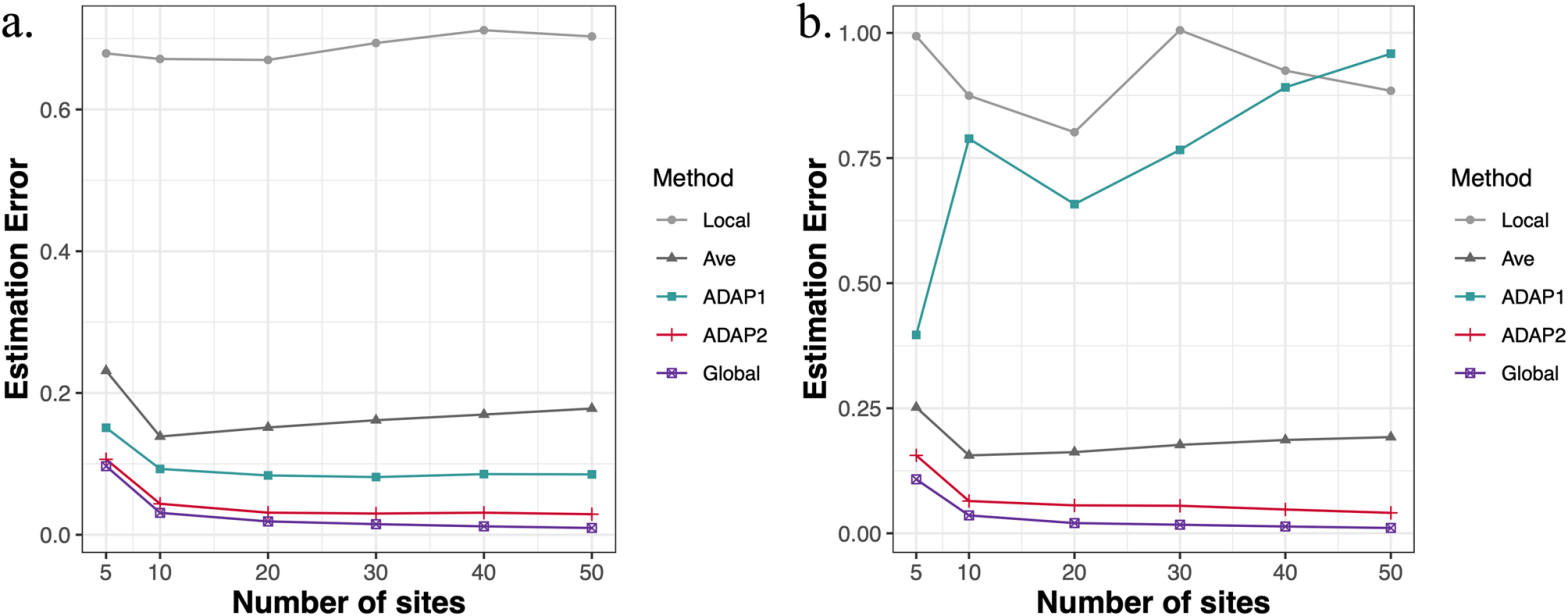
Simulation results under Setting 1 and Setting 2. Both plots display the change of estimation error averaged over 200 replications along with an increasing number of sites *K*. The panel (**a**) is for Setting 1, where covariates are generated from one shared multivariate normal distribution and the panel (**b**) is for Setting 2 where the heterogeneous covariates are considered.

**Figure 2:**
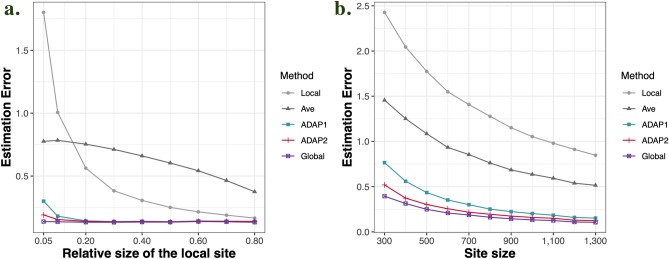
Simulation results under Setting 3 and Setting 4. The panel (**a**) is for Setting 3, which shows the change of estimation error averaged over 200 replications along with an increasing local sample size *n*_1_. The total sample size *N* is fixed at 10,000. The panel (**b**) is for Setting 4 where all sites share the same sample size *n* and the number of sites *K* is fixed at 10. The plot shows the change of estimation error averaged over 200 replications along with an increasing *n* (i.e., an increasing *N*).

Figure 1 displays the results of Setting 1 and Setting 2. As expected, ADAP2 leverages more information from each site’s loss function than ADAP1 and therefore outperforms ADAP1 in terms of estimation error, with an estimation error closest to the pooled estimator among all of the other methods. In particular, we can see in panel (b) of Fig. 1 that heterogeneity in covariates greatly inflates the estimation error of the local estimator and ADAP1 [compared to the homogeneous case displayed in panel (a)], while ADAP2 still maintains a high estimation accuracy, which demonstrates the ability of ADAP2 to handle heterogeneity. In addition, when we increase the number of sites *K*, a larger total sample size $N ( = \sum\nolimits_{k = 1}^{K} {n_{k} } )$ provides both the pooled estimator and the ADAP estimators with more information and improves the estimation accuracy. The average estimator is not as good as the ADAP estimators. Panel (a) of Fig. 2 displays the results of Setting 3, from which we observe that the ADAP methods perform much better than the average estimator and the local estimator which only uses the information from a single site. In terms of the estimation error, ADAP2 is better than ADAP1, especially when the local sample size is small (i.e., $\frac{{n_{1} }}{N} < 0.3$). When $\frac{{n_{1} }}{N}$ gets larger, the ADAP methods perform more similar to the pooled estimator. Thus, in applications, it is preferable to select a lead site with a larger sample size. The results of Setting 4 are displayed in panel (b) of Fig. 2, where $N$ increases along with an increasing *n* and a fixed *K*, and we can see that ADAP methods show a larger improvement over the average and local estimators when *n* is small, and when *n* gets larger the difference between methods becomes smaller. This suggests that using ADAP is the most efficient in a small sample setting.

Tables 1 and 2 display the results of variable selection in Setting 5, and ADAP2 performs better in identifying true positives, but at the expense of having more false positives. The false positive rate of ADAP2 is similar to the pooled estimator and being much lower than the average estimator. Thus, applying ADAP2 ensures a higher probability to recover more true positives than ADAP1 and has a moderate level of false positive rate. The local estimator has a low false positive rate, but its true positive rate is also the lowest among all methods. The average estimator does not perform as well as the ADAP methods since the averaging operation destroys the sparsity of each site’s local estimate.

**Table 1:** True positive rates. The true positive rate is calculated as the percentage (over 400 replications) of coefficients that are estimated to be nonzero among nonzero coefficients in the true coefficient vector.

Magnitude ($\beta_{j}$)	Local	Pooled	Average	ADAP1	ADAP2
0.1	0.14	0.66	0.78	0.59	0.66
0.2	0.39	0.99	0.99	0.96	0.98
0.3	0.61	1.00	1.00	1.00	1.00
0.4	0.75	1.00	1.00	1.00	1.00
0.5	0.84	1.00	1.00	1.00	1.00

**Table 2:** False positive rates. The false positive rate is calculated as the percentage (over 400 replications) of coefficients that are estimated to be nonzero among zero coefficients in the true coefficient vector.

Magnitude ($\beta_{j}$)	Local	Pooled	Average	ADAP1	ADAP2
0.1	0.06	0.12	0.47	0.11	0.16
0.1	0.10	0.14	0.64	0.11	0.18
0.3	0.12	0.15	0.70	0.10	0.18
0.4	0.13	0.15	0.74	0.10	0.17
0.5	0.13	0.16	0.76	0.09	0.16

To account for the uncertainties in the comparison, we have also conducted the one-sided paired *t*-test for each pair of methods to check the significance of the above-claimed benefits. With a significance level of 0.05, the ADAP methods outperform the average and the local estimators in terms of estimation and variable selection for most of the considered settings. The empirical measurements of the improvement and the corresponding test results are displayed in Supplementary Tables 4–9.

### Results from the analysis of the OUD dataset

The logistic lasso regression is applied to the OUD data and fitted by all the above-described methods, and we use site 4 as the lead site without a loss of generality. As the OUD data come from a real distributed research network, they exhibit heterogeneity across sites and provide an ideal environment to compare methods. Therefore, in addition to comparing the estimation and variable selection performance, we also conduct a random-splitting procedure to measure the prediction performance with a set of increasing training set sizes and use AUC as a prediction performance metric for each method.

**Figure 3:**
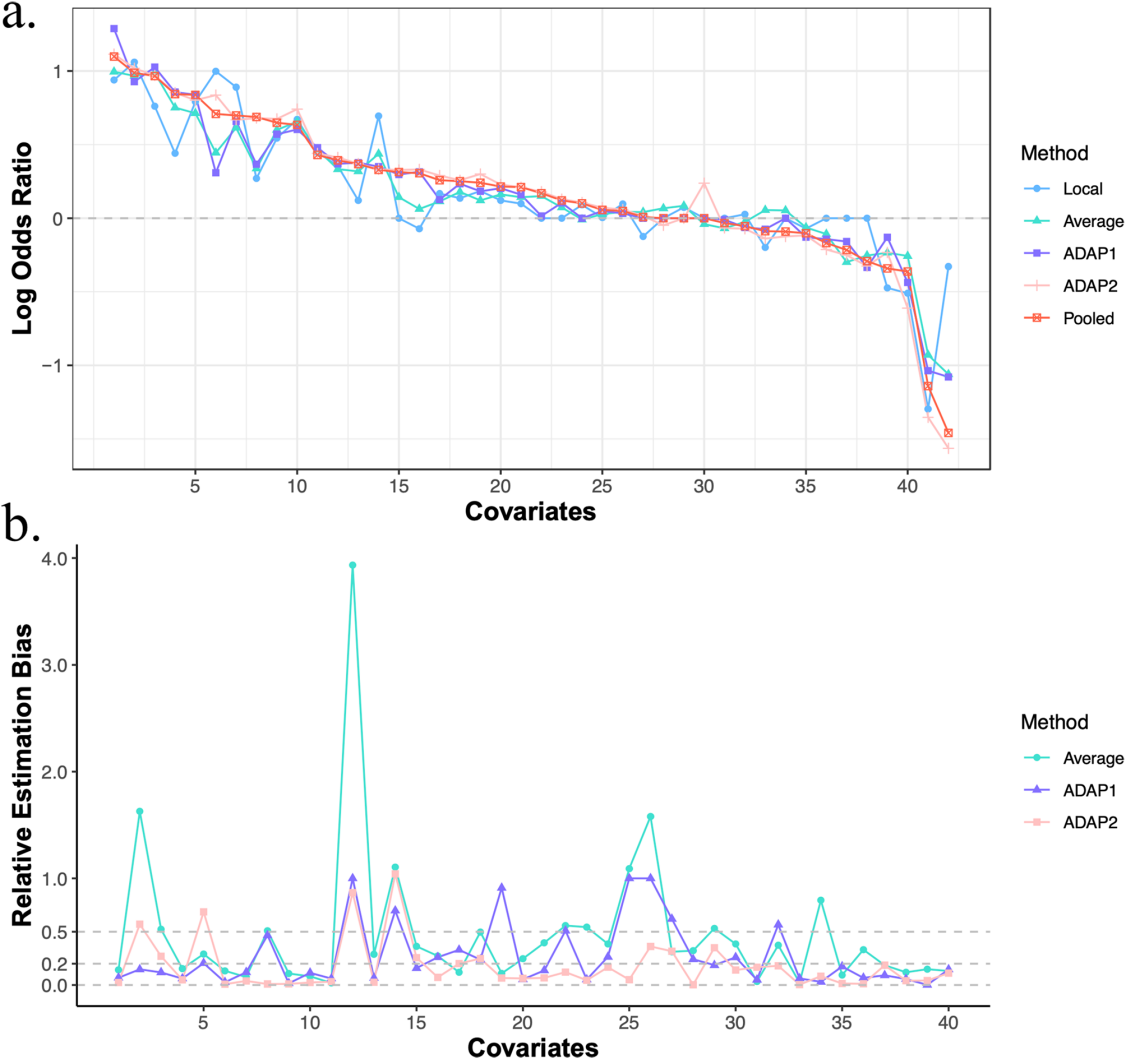
Coefficient estimation results for the OUD analysis. The panel (**a**) displays the coefficient estimates (log odds ratio) for all variables (sorted by the pooled estimates in decreasing order) with the pooled estimator as a gold standard, and the panel (**b**) shows the estimation bias relative to the pooled estimator for all nonzero pooled estimates (without sorting). As the relative estimation bias of the local estimator contains extremely large values, for ease of display we exclude it from panel (**b**). From the two plots, we see that ADAP2 yields estimates that are most consistent with the pooled estimator while the local estimator shows large deviations from the pooled estimator.

It takes around 6 s to fit ADAP1 and takes around 45 s to fit ADAP2 on an iMac with 3.8 GHz 8-Core Intel Core i7 processor, and the estimation results are reported in Supplementary Table 3. As the true coefficient vector is unavailable, we use the pooled estimator $\hat{\beta }_{N}$ as a gold standard and measure the approximation error of other estimators to the gold standard by computing the relative estimation bias with respect to the pooled estimator for each coefficient that has a nonzero pooled estimate (see Fig. 3). Among the methods under comparison, ADAP2 has relative bias < 20% for 73% of the covariates, and it provides the best approximation to the pooled estimator ($\| {\hat{\beta }^{( 2 )} - \hat{\beta }_{N} } \|_{2}^{2} = 0.24$). The local estimator has the largest deviance to the pooled estimator ($\| {\hat{\beta }_{1} - \hat{\beta }_{N} } \|_{2}^{2} = 2.72$), and only 25% of the covariates have relative bias < 20%. The average estimator does not perform as well as the ADAP methods ($\| {\hat{\beta }_{ave} - \hat{\beta }_{N} } \|_{2}^{2} = 0.88$) and has 40% of the covariates having relative bias < 20%. ADAP1 has 60% of the covariates whose relative bias < 20% and $\| {\hat{\beta }^{( 1 )} - \hat{\beta }_{N} } \|_{2}^{2} = 0.64.$ Thus, among the four methods in this analysis, ADAP2 is the most consistent with the pooled estimator. ADAP1’s performance is better than the local and the average estimators, which can be explained by the relatively homogeneous covariate distributions among the five sites (see Supplementary Table 2).

As for variable selection, by treating the pooled estimator as a gold standard, the local estimator and average estimator each have three estimates whose signs are opposite to the corresponding pooled estimates. The local estimator has one false positive while the average estimator and ADAP2 estimator have three and two false positives, respectively. The local estimator missed nine predictive covariates and the ADAP1 estimator missed three predictive covariates. In view of these considerations, ADAP2 performs better than the other methods.

**Figure 4:**
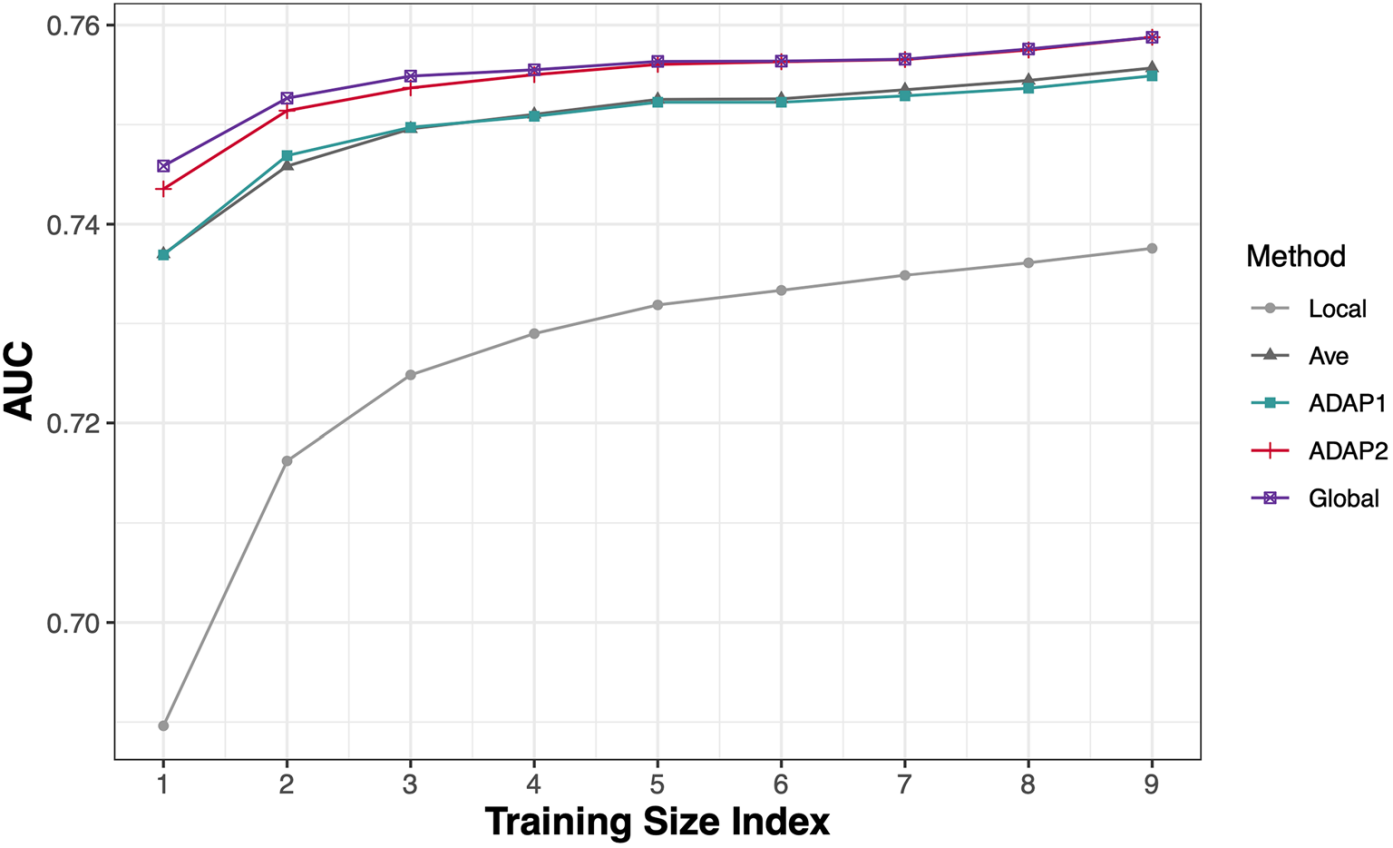
Prediction performance measured by averaging AUCs obtained through 200 random-splitting procedures on the OUD data. The training size index $t$ takes values from 1 to 9, and each *t* means that we randomly select $t \times 100$ cases and $t \times 200$ controls from each site to form a training set, with the remaining data used to test the fitted model. Thus, the plot shows the pattern of variation in AUC as a function of the size of the training set. In general, as the training set size becomes larger, the AUC is increased for all methods. Among the five methods, the pooled estimator performs the best, with the performance of ADAP2 very close to that of the pooled estimator. ADAP1 and the average estimator have lower AUCs than ADAP2, and the local estimator has the worst prediction performance.

We present the prediction results in Fig. 4. In general, as the training set becomes larger, the AUC increases for all methods. Among the five methods, the pooled estimator has the best prediction performance and ADAP2 performs very close to the pooled estimator. ADAP1 and the average estimator have lower AUCs than ADAP2, and the local estimator has the worst prediction performance. We have constructed the empirical 95% confidence intervals defined by the 2.5th percentile and the 97.5th percentile of the pairwise AUC difference between ADAP2 and the local, the average, and ADAP1 estimator to check the significance of the prediction performance improvement for ADAP2. Once the confidence interval is on the positive side of zero, there is a significant improvement. The results are in Supplementary Table 10 and show a significant improvement for most cases, and for the remaining cases, ADAP2 performs as well as other methods. Thus, by collecting the second-order gradient information from all sites to form the surrogate function, ADAP2 has a comparable or higher prediction accuracy than other methods and performs as well as the pooled estimator.

Based on the estimated effects, for the patients with chronic pain and an opioid prescription, some co-occurring psychiatric and substance use disorder diagnoses (e.g., anxiety and cocaine-related disorder) contribute to the identification of OUD-positive cases. Interestingly, patients with sleep disorders are at decreased risk of OUD. Compared to patients with a normal BMI, patients who are overweight or obese are less likely to be OUD positive. Smoking is a risk factor for OUD, and non-Hispanic white (NHW) patients have a greater risk to develop OUD. People receiving Medicaid also have a higher risk of being OUD positive. Age and sex also have effects on the risk of OUD, with adults younger than 64 and men at greater risk of the disorder.

## Discussion

In this study, we introduce a one-shot privacy-preserving algorithm to fit penalized regression in a distributed manner. A properly selected penalty on the coefficient vector is added to the surrogate loss function to balance the trade-off between goodness-of-fit and model complexity. The first-order and second-order gradients are collected from collaborating sites and contribute to construction of the surrogate loss function. The simulation study and the application to the analysis of OUD data both demonstrate the superiority of the ADAP methods over the local and average estimators. Moreover, the improvement of ADAP2 over ADAP1 is achieved at the expense of transferring more digits to the lead site, i.e., transferring the second-order gradient information that contains $O( {Kp^{2} } )$ numbers. With a moderate dimension $p$, transferring $O( {Kp^{2} + Kp} )$ numbers will not result in severe technical problems in practical applications. However, this becomes a limitation of ADAP2 when the dimension *p* goes large and new techniques are then needed to reduce the communication burden. Otherwise, using ADAP1 is preferable in this situation.

Iterative algorithms are promising to further improve the estimation accuracy of ADAP methods^19^. However, the number of digits transferred in the iterative communications could be significantly larger than one-shot algorithms, potentially increasing the risk of re-identification of subjects. Moreover, in practice, the higher communication cost, specifically more rounds of communications involving several different requests made by researchers to each individual site participating in a study, also leads to higher operational efforts across participating sites, which requires timely cooperation and coordination among the researchers and could potentially delay the completion of the learning projects. In contrast, one-shot methods are more appealing as they do not require iterative communication. Specifically, in ADAP, after the initial estimator being broadcasted from the lead site to the collaborating sites, the collaborating sites only need to take one round of action, i.e., receive the broadcasted estimates, calculate and transfer the gradient information to the lead site, which saves both time and labor cost.

Following Friedman et al.^41^, we adopted the coordinate descent algorithm within a nested loop together with 5-fold cross-validation to fit a solution path and select the tuning parameter. We also experimented with using the lead site’s tuning parameter to fit the surrogate model, but the resultant estimator approximated the pooled estimator poorly. The *l*_1_ penalty introduces bias into the estimates when it filters off random noise, i.e., it pushes all the coefficients toward zero, leaving only those with strong signals, but shrunken in magnitude. When the sample is large enough and the signal is strong, a small tuning parameter is preferred; otherwise, a larger tuning parameter is needed to recover the true signals from the random noise. Therefore, with a large difference in the amount of available information, the magnitude of regularization needed by a local model is too large for the global model. Another concern in tuning is the transferability of the algorithm, as the tuning is done at the lead site. By changing to a different lead site, the final model could be different in the selected covariates and its estimates, and this is also a limitation of ADAP and requires further investigation. Some of distributed algorithms that employ the same surrogate loss function strategy select each site as a lead site and then average all of the resultant estimators as a final estimator. However, this is not applicable in our case, as taking an average can degrade the variable selection. In practice, to avoid this effect and achieve a better approximation to the pooled model, we recommend using a lead site with a large sample.

A practical concern in distributed learning is the potential heterogeneity across multiple sites, including the heterogeneity underlying the association pattern (as each site has model parameters that are not exactly the same) and the covariate distribution. There are existing efforts devoted to solving the heterogeneity problem in distributed learning. For example, Tong et al.^28^ used the robustness of the median compared to the mean to relieve the effects of ‘outlying studies’, Duan et al.^29^ proposed a density ratio tilting method to accommodate the heterogeneous nuisance parameters across sites, and Cai et al.^42^ employed effect decomposition to allow site-specific effects of covariates on the outcome. Another limitation of ADAP is that ADAP2 only handles the heterogeneity in covariate distributions by incorporating the second-order gradients of all sites when creating the surrogate objective function. Therefore, further development of ADAP methods to deal with heterogeneity in the model parameters is needed.

Some important work remains for future investigation. First, as the number of high-dimensional association studies increases (e.g., in genome-wide association studies that connect a phenotype with millions of genotypes to identify risk variants), methods are needed to reduce the communication cost of sharing second-order gradients. Some numerical methods that approximate the second-order gradient provide a possible solution that warrants further exploration. Another pressing issue that requires resolution is the heterogeneity in model parameters. Whereas a mixed-effects model could be used in this situation^10,43^, we plan to explore this and other modeling approaches^44,45^ in the high-dimensional scenario to develop distributed algorithms that integrate information across multiple datasets while accounting for heterogeneity. Generalization of our work to other types of outcomes such as count data^46^ is of interest. Third, we regard ADAP as a privacy-preserving approach since it is a one-shot distributed algorithm where only summary statistics are needed to communicate across sites participating in a collaborative study. However, we have not rigorously checked if ADAP meets some privacy-preserving criteria such as *k*-anonymity^47^ or differential privacy^48,49^. In the future, we will measure the privacy leaking risk of applying ADAP and enhance it by using some techniques such as differential privacy and multiparty homomorphic encryption^50^. Finally, to evaluate the portability of predictive models constructed via the ADAP algorithm using data from OneFlorida data, we plan to evaluate them externally using the data from the STAR (Stakeholders, Technology, and Research) Network, which contains centralized data from 8 healthcare organizations.

To conclude, in this study we considered a communication-efficient distributed learning framework for penalized regression. There are multiple penalty functions and regression models that can be embedded into this framework to satisfy different analysis demands. Simulation studies and an application to a clinical research network studying OUD demonstrated the validity and feasibility of the ADAP methods. The novelty of the proposed method mainly manifests in the following aspects. First, by exploiting the surrogate likelihood idea, ADAP provides a flexible framework to apply penalized regression distributedly. The algorithm protects the patients’ privacy and only requires one round of communication from the collaborating site. Second, by constructing a surrogate of the global likelihood function, ADAP outperforms the average-type estimators in that it accommodates rare outcomes and small sample sizes, and it also avoids severe over-selection brought by the averaging operation. Third, compared with some methods that are not robust to the violation of the homogeneity assumption, incorporation of the second-order gradient information in ADAP2 successfully accommodates heterogeneity in covariate distributions while boosting the true positive rate and improving estimation and prediction accuracy. We conclude that the ADAP2 estimator is a good approximation of the pooled estimator that is robust to heterogeneity across sites. The application of these findings to OUD underscores the potential contribution of this approach for addressing important public health problems.

## Methods

### Data and problem formulation

We consider the relationship between an outcome $y \in R$ and a set of covariates $x = ( {1,x_{1} , \ldots ,x_{p - 1} } )^{T} \in R^{p}$. Suppose in a multisite study with *K* sites, we observe $( {x_{ki} , \;y_{ki} } ), \; i = 1, \ldots ,n_{k}$ for the *i*-th individual in the *k*-th site $k = 1, \ldots ,K$, where $\sum\nolimits_{k = 1}^{K} {n_{k} = N}$ is the total sample size. We model $P( {y{|}x} )$ using a regression model with coefficient $\beta$, and a loss function in the *k*-th site is defined as

$$L_{k} ( \beta ) = \frac{1}{{n_{k} }}\mathop \sum \limits_{i = 1}^{{n_{k} }} f( {\beta ;\;x_{ki} ,\;y_{ki} } ) ,$$

where $f( {\beta ;\;x_{ki} ,\;y_{ki} } )$ is a prespecified loss function. For example, $f( {\beta ;\;x_{ki} ,\;y_{ki} } ) = - y_{ki} x_{ki}^{T} \beta + \log \{ {1 + \exp ( {x_{ki}^{T} \beta } )} \}$ if we use a logistic regression. We first define several benchmark estimators.

### Benchmark methods

Without loss of generality, we let site 1 be the lead site and obtain the **local estimator** as

$$\hat{\beta }_{1} = \arg \mathop {\min }\limits_{{\upbeta }} L_{1} ( \beta ) + P_{\lambda } ( \beta )\quad \quad ({\text{local}}),$$

where $P_{\lambda } ( \beta )$ is a penalty function with $\lambda > 0$ being a tuning parameter controlling the level of penalization. Similarly, for other sites we can obtain $\hat{\beta }_{k} = \arg \mathop {\min }\limits_{{\upbeta }} L_{k} ( \beta ) + P_{\lambda } ( \beta )$ based on locally stored data, and the **average estimator** can then be defined as the weighted average of each site’s estimate

$$\hat{\beta }_{ave} = \mathop \sum \limits_{k = 1}^{K} \frac{{n_{k} }}{N}\hat{\beta }_{k} \quad \quad ( {{\text{average}}} ).$$

By assuming that individual-level data-sharing is allowed, we obtain the global loss function

$$L( \beta ) = \frac{1}{N}\mathop \sum \limits_{k = 1}^{K} n_{k} L_{k} ( \beta ),$$

and get the **pooled estimator** from

$$\hat{\beta }_{N} = \arg \mathop {\min }\limits_{{\upbeta }} L( \beta ) + P_{\lambda } ( \beta )\quad \quad ( {{\text{pooled}}} ).$$

The local estimator is not efficient as it does not utilize information from other sites. The pooled estimator is considered as a gold standard as it directly uses all the patient-data without constrains on data-sharing, but it is not available in practice. We then introduce ADAP estimators.

### Proposed method: ADAP

The main idea of ADAP is to build a surrogate loss function^19,20^ to approximate $L( \beta )$ as

$$\tilde{L}^{1} ( \beta ) = L_{1} ( \beta ) + \{ {\nabla L( {\overline{\beta }} ) - \nabla L_{1} ( {\overline{\beta }} )} \}^{T} \beta$$

where $\nabla L_{k} ( {\overline{\beta }} ) = \frac{1}{{n_{k} }}\sum\nolimits_{i = 1}^{{n_{k} }} {\nabla f} ( {\overline{\beta };\;x_{ki} ,\;y_{ki} } )$ denotes the first-order gradient of $L_{k} ( \beta ){ }$ at an initial estimator $\overline{\beta }$, and $\nabla L( {\overline{\beta }} ) = \frac{1}{N}\sum\nolimits_{k = 1}^{K} {n_{k} \nabla L_{k} ( {\overline{\beta }} )}$. In this way, each collaborating site only needs to send its first-order gradient (a *p*-dimensional vector) to the lead site to construct $\tilde{L}^{1} ( \beta )$. The **ADAP1 estimator**^19,20,27^ is obtained from

$$\hat{\beta }^{( 1 )} = \arg \mathop {\min }\limits_{{\upbeta }} \tilde{L}_{1} ( \beta ) + P_{\lambda } ( \beta )\quad \quad ( {{\text{ADAP1}}} ).$$

This approximation can be improved by further requiring the second-order gradient of $L_{k} ( \beta )$, which gives us the second-order surrogate function^22^

$$\tilde{L}^{2} ( \beta ) = L_{1} ( \beta ) + \{ {\nabla L( {\overline{\beta }} ) - \nabla L_{1} ( {\overline{\beta }} )} \}^{T} \beta + \frac{1}{2}( {\beta - \overline{\beta }} )^{T} \{ {\nabla^{2} L( {\overline{\beta }} ) - \nabla^{2} L_{1} ( {\overline{\beta }} )} \}( {\beta - \overline{\beta }} )$$

where $\nabla^{2} L_{k} ( \beta ) = \frac{1}{{n_{k} }}\sum\nolimits_{i = 1}^{{n_{k} }} {\nabla^{2} f( {\overline{\beta };\;x_{ki} ,\;y_{ki} } )}$ is the second-order gradient (a $p \times p$-dimensional matrix) and $\nabla^{2} L( {\overline{\beta }} ) = \frac{1}{N}\sum\nolimits_{k = 1}^{K} {n_{k} \nabla^{2} L_{k} ( {\overline{\beta }} )}$. It is worth mentioning that, by collecting the second-order gradients from all sites, the resulting estimator is robust to the potential heterogeneity in covariate distributions. Therefore, at the expense of transferring additional $O( {Kp^{2} } )$ numbers, we can construct $\tilde{L}^{2} ( \beta )$ and get the **ADAP2 estimator.**

$$\hat{\beta }^{( 2 )} = \arg \mathop {\min }\limits_{{\upbeta }} \tilde{L}_{2} ( \beta ) + P_{\lambda } ( \beta )\quad \quad ({\text{ADAP2}}).$$

To satisfy various demands, different penalties can be applied, e.g., $P_{\lambda } ( \beta ) = \lambda \| \beta \|_{1}$ for the lasso regression and $P_{\lambda } = \lambda ( {\alpha \| \beta \|_{1} + ( {1 - \alpha } )\| \beta \|_{2}^{2} } )$ with $\alpha \in ( {0,\;1} )$ for the elastic-net method. For illustration, in the following we mainly consider the lasso logistic regression. As for selecting the initial estimator $\overline{\beta }$, both the local estimator $\hat{\beta }_{1}$ and the average estimator $\hat{\beta }_{ave}$ are good choices. The algorithm for both ADAP1 and ADAP2 is summarized below.
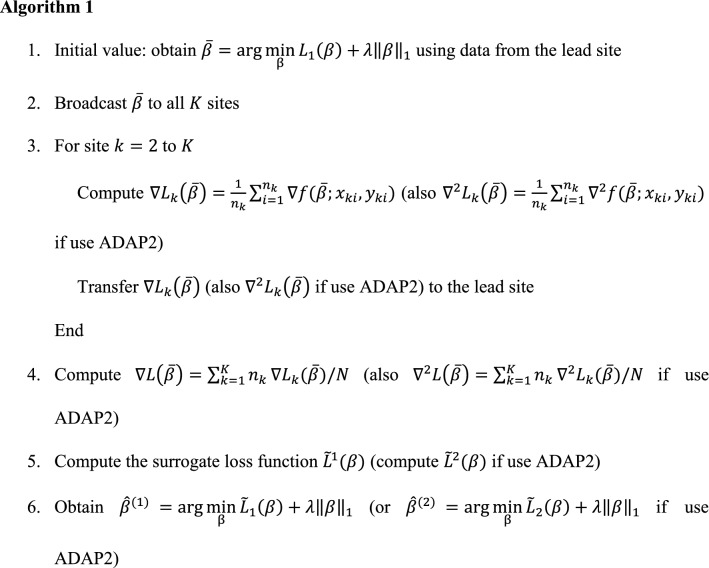


We note that in Step 6, a solution path of ADAP is obtained by embedding the coordinate descent algorithm into a nested loop^41^, and we use a modified 5-fold cross-validation to select $\lambda$. Specifically, each time we leave out a fold of local data and use the remaining four folds together with the aggregated gradient information to form a surrogate loss function and obtain a solution path, and then we use the left-out data to compute the deviance, i.e., the negative log-likelihood function evaluated at the current estimates. The selected $\lambda$ is the one that minimizes the averaged deviance among 5 folds.

Algorithm 1 requires only one round of communication from the collaborating sites to the lead site to transfer the gradient information. In total $O( {Kp} )$ and $O( {Kp + Kp^{2} } )$ numbers are transferred in ADAP1 and ADAP2, respectively. For comparison among methods, the computation based on the global loss function requires sharing individual-level data (i.e., $O( {KNp} )$ numbers) and is prohibited in practice due to privacy concerns; the local loss function does not need data-sharing; the average estimator requires collaborating sites to share their estimates (in total $O( {Kp} )$ numbers) to the lead site in one round of communication. As shown in our simulation and real-world data application, although the communication cost is comparable to that of the average estimator (for ADAP1) or greater than the average estimator (for ADAP2), ADAP algorithms perform better than the average estimator.

### Simulation study

We evaluate the performance of ADAP under five settings, and the methods under comparison are: the local estimator $\hat{\beta }_{1}$, the average estimator $\hat{\beta }_{ave} ,$ the pooled estimator $\hat{\beta }_{N}$, ADAP1 estimator $\hat{\beta }^{( 1 )}$, and ADAP2 estimator $\hat{\beta }^{( 2 )}$. Without a loss of generality, site 1 is treated as the lead site. The considered settings are:

- **Setting 1**: We fix the local sample size $n_{1} = 1,000$ and increase the number of sites $K \in ( {5, \;10, \;20, \;30, \;40, \;50} )$. For other sites, we let $n_{k} = 1,000 \times 10^{{u_{k} }}$ with $u_{k} \sim U( { - 1,\;1} )$. The dimension is $p = 200$ and the covariates are generated from a multivariate normal distribution $z_{ki} \sim N_{p - 1} ( {0,\;{\Sigma }} )$ with ${\Sigma } = ( {0.1^{{I( {i \ne j} )}} } ) \in R^{{( {p - 1} ) \times ( {p - 1} )}}$ and $x_{ki} = ( {1,z_{ki}^{T} } )^{T}$. The true coefficient vector $\beta^{*} =( { - 2.5,\;\underbrace {0.5, \ldots , 0.5}_{10},\;\underbrace {0, \ldots , 0}_{189} } )$.
- **Setting 2**: The covariates are generated from heterogeneous multivariate normal distributions, i.e., $z_{ki} \sim N_{p - 1} ( {\mu_{k} ,\;{\Sigma }_{{\text{k}}} } )$ where $\mu_{k} = ( {\mu_{k1} ,\;\underbrace {0, \ldots 0}_{p - 2}}) \in R^{p - 1}$ with $\mu_{k1} \sim U( { - 1, \;1} )$, ${\Sigma }_{{\text{k}}} = ( {\rho_{k}^{{I( {i \ne j} )}} } ) \in R^{{( {p - 1} ) \times ( {p - 1} )}}$ with $\rho_{k} \sim U( {0.1, \;0.5} )$ and $x_{ki} = ( {1,\;z_{ki}^{T} } )^{T}$. We let $\mu_{11} = \mathop {\min }\limits_{k} \mu_{k1}$, and if $z_{1}$ is the age, then this setting is to mimic the scenario where the lead site’s patients are the youngest among all the sites. All other details are the same as in setting 1.
- **Setting 3**: We fix the total sample size *N* = 10,000, and let $K = 11$ and increase the local sample size $n_{1} \in ( {500,\; 1,000, \;2,000,\; 3,000, \;4,000, \;5,000, \;6,000, \;7,000, \;8,000} ).$ The remaining $N - n_{1}$ samples are evenly assigned to other sites. The dimension is $p = 200,$ and the covariates are generated as in setting 1 except ${\Sigma } = ( {0.5^{{I( {i \ne j} )}} } ) \in R^{{( {p - 1} ) \times ( {p - 1} )}}$. The true coefficient vector is $\beta^{*} = ( { - 2, \;1.5, \;1,\; 1,\;1,\;1,\;\underbrace {0, \ldots 0}_{194}} ).$
- **Setting 4**: We fix the number of sites $K = 10$, and let $n_{k} = n$ for $k = 1,2, \ldots K$ and increase the sample size $n \in ( {300,\; 400, \;500, \ldots ,\;1,300} )$. All other details are the same as in setting 1 except ${\Sigma } = ( {0.5^{{I( {i \ne j} )}} } ) \in R^{{( {p - 1} ) \times ( {p - 1} )}}$.
- **Setting 5**: This setting is to see the variable selection performance of the ADAP methods. In total we have 10 sites and each site has 1000 samples. The dimension is $p = 200$, and the covariates are generated as in setting 1 except ${\Sigma } = ( {0.5^{{I( {i \ne j} )}} } ) \in R^{{( {p - 1} ) \times ( {p - 1} )}}$. We let the true intercept to be -2 and let 10 covariates have the same non-zero coefficients with magnitude selected from (0.1, 0.2, 0.3, 0.4, 0.5). All the other coefficients are zero.

For the first four settings, the simulation is repeated 200 times and the number of replications under Setting 5 is 400. We calculate the Euclidean distance of the estimate to its true value to see the parameter estimation performance, e.g., the estimation error for the local estimator is calculated as $\frac{1}{200}\mathop \sum \nolimits_{r = 1}^{200} \| { \hat{\beta }_{1}^{( r )} - \beta^{*} } \|_{2}$, and we use the true positive rate and false positive rate to see the variable selection performance for Setting 5.

### Application to opioid use disorder (OUD) data

The logistic lasso regression is applied to data from five participating sites of the OneFlorida Clinical Research Consortium^30^. Combining all five sites, we obtained a total of 5000 cases and 10,000 controls, with each site contributing a small subset of all of their EHRs, i.e., 1000 cases and 2000 controls, or a case–control ratio of 1:2. A list of risk factors was compiled from the literature and extracted from the database, including basic demographic features such as age, gender and race, and co-occurring diagnoses, e.g., depression and sleep disorder (see Supplementary Table 2 for all covariates).

For smoking status, race, and insurance type, we treat missing values as a separate group. BMI (body mass index, taking the average value during the 12 months before the first prescription) and age are categorized into discrete variables to model the possible nonlinear relationship between these factors and OUD. As there is a large proportion of missing values (52.3%) for BMI, we imputed them using an R package MICE^51^ by regressing BMI on all the other variables in the data and then predicting the missing values based on the fitted model. We then categorized them based on a pre-specified range stated in Supplementary Table 1, where the details of all the dummy variable creation can be found. After excluding nine extremely rare (with a prevalence in the overall sample < 0.2%) covariates from the analysis, we have 42 covariates in the model. See Supplementary Table 2 for a summary of characteristics of each variable across the five sites.

Without a loss of generality, we use site 4 as the lead site and apply all the above-described methods to the OUD data. In addition to comparing the estimation and variable selection performance, we conduct a random-splitting procedure to measure the prediction performance. Specifically, we randomly decompose the whole dataset into a training set to fit the model and a testing set to calculate AUC. Using a training size index $t$ ($t = 1, \ldots ,9$) to denote the training set size, each time we randomly select $t \times 100$ cases and $t \times 200$ controls from each site to form a training set and use the remaining samples as the testing set. To account for the randomness of decomposition, we repeat the random-splitting procedure 200 times and compute the average AUC for each method as a prediction performance metric. Note that when $t$ is small, there are several fittings that failed due to the existence of some rare covariates whose prevalence becomes even lower or achieves zero in the training set after data splitting. To ensure more robust results, the three least prevalent covariates are removed, and the average AUC is calculated based on the successfully fitted results.

### Ethics

The experimental protocol was approved by the University of Florida (UF) Institute Review Board (IRB) as the ethics committee under the protocol number IRB202001100. As part of the UF IRB process, the protocol has been reviewed in accordance with the institutional guidelines and consent waivers were approved as part of the IRB protocol.

## Supplementary Information

Supplementary Information.

## Data Availability

The OUD dataset is available upon application to the OneFlorida+ network through the link: https://onefloridaconsortium.org/front-door/research-infrastructure-utilization-application/. For the reader’s convenience, a synthetic OUD dataset and the related R codes to conduct analysis can be found at https://github.com/Penncil/ADAP.

## Code availability

For dissemination, the R codes are available at https://github.com/Penncil/pda and through our R package: ‘pda’^52^ in version 1.0-2.
