# Supplementary material for: Multisite learning of high-dimensional heterogeneous data with applications to opioid use disorder study of 15,000 patients across 5 clinical sites
Source: Sci Rep. 2022 Jun 30;12:11073. doi: 10.1038/s41598-022-14029-9 (PMC9245877; doi:10.1038/s41598-022-14029-9)
Supplement: Supplementary file 1 — Supplementary Information. [file 41598_2022_14029_MOESM1_ESM.pdf]

Supplementary Material for "Multisite learning of high-dimensional heterogeneous data with applications to opioid use disorder study of 15,000 patients across 5 clinical sites"

Authors

Xiaokang Liu, PhD[1]
Rui Duan, PhD[2]
Chongliang Luo, PhD[1,3]
Alexis Ogdie, MD, MSCE[1]
Jason H. Moore, PhD[1]
Henry R. Kranzler, MD[4]
Jiang Bian, PhD[5]
Yong Chen, PhD[1*]

* Corresponding author

Affiliation of the authors:

[1]Department of Biostatistics, Epidemiology and Informatics, University of Pennsylvania Perelman School of Medicine, Philadelphia, PA, USA
[2]Harvard T.H. Chan School of Public Health, Harvard University, Boston, MA, USA
[3]Division of Public Health Sciences, Washington University School of Medicine in St. Louis, St. Louis, MO, USA
[4]Department of Psychiatry, University of Pennsylvania Perelman School of Medicine and the VISN 4 MIRECC, Crescenz VAMC, Philadelphia, PA, USA
[5]Department of Health Outcomes and Biomedical Informatics, University of Florida Health Cancer Center, Gainesville, FL, USA

Correspondence:

Yong Chen, PhD
University of Pennsylvania Perelman School of Medicine
423 Guardian Drive
Philadelphia, PA 19104
Office: 215-746-8155
E-mail: ychen123@upenn.edu

Supplemental Table 1. Dummy variable definition. We use “ref” to denote reference groups.

	**var**		**var**		**var**
**Age**		**BMI_avg**		**Race/Ethnicity**	
< 18	Age_1	9.5 <= & < 18.5	bmi_1	NHW	Race_1
18 <= & < 39	Age_2	18.5<= & < 25 (ref)		Others (ref)	
39 <= & < 64	Age_3	25 <= & < 30	bmi_2	Unknown	Race_2
>= 65 (ref)		30 <= & <= 90	bmi_3		
				**Insurance Type**	
**Sex**		**Smoking**		MEDICAID	Insu_1
Female	Gender_1	Yes	Smoke_1	Others (ref)	
Male (ref)		No (ref)		Unknown	Insu_2
		Missing	Smoke_2		

Supplemental Table 2. Characteristics of all 42 covariates across five sites. All covariates are coded as binary variables, and the presented numbers are their prevalence (%) in each site.

Covariates	Site 1	Site 2	Site 3	Site 4	Site 5
**alcohol_related_disorders**	1.73	2.60	2.57	2.30	2.57
**depression**	7.40	8.70	12.17	10.90	11.27
**anxiety**	11.17	14.00	14.97	13.53	11.07
**sleep_disorder**	4.43	3.23	7.47	4.97	5.97
**rheumatoid_arthritis**	1.93	1.80	1.93	1.50	2.10
**pain**	14.40	23.63	12.43	17.00	12.70
**cannabis_related_disorder**	1.20	2.97	2.23	2.07	1.93
**sedative_related_disorder**	0.40	0.57	0.23	0.27	0.63
**cocaine_related_disorder**	0.67	2.80	1.73	1.27	2.77
**nicotine_related_disorder**	11.50	16.13	15.17	20.67	11.30
**other_psychoactive_disorder**	1.80	1.90	1.43	2.13	2.67
**CCI_Myocardial_infarction**	2.67	3.30	3.03	2.10	2.27
**CCI_Congestive_heart_failure**	4.57	3.80	5.63	5.00	4.50
**CCI_Peripheral_vascular_disease**	3.37	3.33	4.70	4.63	5.33
**CCI_Cerebrovascular_disease**	3.07	2.73	4.83	4.73	3.87
**CCI_Dementia**	0.70	0.53	0.63	0.57	1.00
**CCI_Chronic_pulmonary_disease**	15.20	16.57	15.93	16.07	13.73
**CCI_Rheumatic_disease**	3.13	2.83	2.43	2.13	3.40
**CCI_Peptic_ulcer_disease**	1.17	1.00	0.73	0.97	1.00
**CCI_Mild_liver_disease**	3.90	3.90	2.87	5.57	6.00
**CCI_Diabetes_without_chronic_complication**	15.67	13.00	13.27	12.97	15.60
**CCI_Diabetes_with_chronic_complication**	3.43	2.73	3.13	3.23	5.13
**CCI_Hemiplegia_or_paraplegia**	0.93	1.00	1.37	1.63	2.40
**CCI_Renal_disease**	4.60	3.83	4.40	3.50	5.23
**CCI_Any_malignancy**	0.47	1.20	0.33	1.33	0.20
**CCI_Moderate_or_severe_liver_disease**	0.47	0.60	0.23	0.80	0.83
**CCI_AIDS_HIV**	0.33	0.77	1.03	1.07	2.67
**insomia**	0.57	0.47	1.03	1.53	2.00
**sleep_apnea**	3.77	2.80	6.60	3.63	3.77
**bmi_1**	3.20	3.40	4.03	4.17	3.17
**bmi_2**	29.67	27.33	29.03	29.47	28.93
**bmi_3**	41.90	45.10	40.93	38.40	40.13
**smoke_1**	5.37	8.07	25.13	28.90	0.17
**smoke_2**	88.67	90.83	52.80	42.23	99.33
**race_1**	53.93	49.63	64.57	65.60	13.87
**race_2**	0.93	1.13	4.50	1.40	4.20
**insu_1**	24.93	22.43	32.87	41.73	45.37
**insu_2**	57.77	2.37	1.90	14.67	20.70
**age_1**	3.00	2.90	4.60	6.10	2.80
**age_2**	38.43	49.67	33.00	34.37	24.87
**age_3**	41.67	35.63	43.67	45.67	54.23
**gender_1**	64.13	70.57	63.23	61.30	57.50

Note: The Charlson Comorbidity Index (CCI) is calculated using ICD codes from each individual's medical history [1]. The conditions included in the CCI are myocardial infarction, congestive heart failure, cerebrovascular disease, dementia, chronic pulmonary disease, rheumatologic disease, peptic ulcer disease, mild liver disease, diabetes, diabetes with chronic complications, hemiplegia or paraplegia, renal disease, any malignancies, moderate or severe liver disease, metastatic solid tumors, and AIDS. The diagnostic codes and calculation of CCI were performed as described by Deyo et al. [2] and Quan et al. [3]

Supplemental Table 3. The coefficient estimates of all 42 covariates given by the local estimator, the average estimator, the ADAP1 estimator, the ADAP2 estimator and the pooled estimator. Compared to the pooled estimator, all the discordant estimates obtained by other methods are highlighted. Specifically, the estimates that are discordant in signs are marked in blue, the false positives are marked in red, and false negatives are marked in yellow.

Covariates	Local	Average	ADAP1	ADAP2	Pooled
**(Intercept)**	-3.28	-2.55	-2.77	-3.04	-2.97
**alcohol_related_disorders**	-0.20	0.06	-0.08	-0.14	-0.09
**depression**	0.00	0.03	0.05	0.07	0.06
**anxiety**	0.35	0.33	0.37	0.41	0.39
**sleep_disorder**	-0.51	-0.26	-0.44	-0.61	-0.36
**rheumatoid_arthritis**	0.00	0.07	0.00	-0.05	0.00
**pain**	0.12	0.32	0.38	0.37	0.37
**cannabis_related_disorder**	0.54	0.60	0.57	0.67	0.65
**sedative_related_disorder**	0.27	0.34	0.37	0.68	0.69
**cocaine_related_disorder**	0.44	0.75	0.86	0.85	0.84
**nicotine_related_disorder**	0.45	0.47	0.48	0.44	0.43
**other_psychoactive_disorder**	1.06	0.97	0.93	1.02	0.99
**CCI_Myocardial_infarction**	-0.12	0.04	0.00	0.02	0.01
**CCI_Congestive_heart_failure**	0.14	0.18	0.23	0.26	0.25
**CCI_Peripheral_vascular_disease**	0.00	-0.07	-0.01	-0.07	-0.03
**CCI_Cerebrovascular_disease**	0.00	-0.11	-0.14	-0.21	-0.17
**CCI_Dementia**	-0.33	-1.06	-1.08	-1.56	-1.46
**CCI_Chronic_pulmonary_disease**	0.10	0.04	0.03	0.06	0.05
**CCI_Rheumatic_disease**	0.18	0.12	0.18	0.30	0.24
**CCI_Peptic_ulcer_disease**	0.00	0.15	0.01	0.18	0.17
**CCI_Mild_liver_disease**	0.12	0.16	0.20	0.23	0.21
**CCI_Diabetes_without_chronic_complication**	0.00	0.07	0.10	0.13	0.12
**CCI_Diabetes_with_chronic_complication**	0.07	0.08	0.00	0.00	0.00
**CCI_Hemiplegia_or_paraplegia**	0.17	0.11	0.13	0.29	0.26
**CCI_Renal_disease**	0.00	0.14	0.30	0.33	0.31
**CCI_Any_malignancy**	0.00	-0.30	-0.16	-0.25	-0.22
**CCI_Moderate_or_severe_liver_disease**	0.09	-0.01	0.00	0.11	0.10
**CCI_AIDS_HIV**	0.00	0.05	0.00	-0.12	-0.09
**insomia**	-0.47	-0.24	-0.13	-0.23	-0.34
**sleep_apnea**	0.00	-0.04	0.00	0.24	0.00
**bmi_1**	0.10	0.14	0.16	0.21	0.21
**bmi_2**	0.03	-0.03	-0.06	-0.07	-0.05
**bmi_3**	-0.07	-0.06	-0.13	-0.12	-0.10
**smoke_1**	0.67	0.66	0.60	0.74	0.63
**smoke_2**	1.00	0.44	0.31	0.84	0.71
**race_1**	0.76	0.99	1.03	0.97	0.97
**race_2**	-0.07	0.06	0.32	0.33	0.31
**insu_1**	0.94	1.00	1.29	1.11	1.10
**insu_2**	0.69	0.44	0.35	0.33	0.33
**age_1**	-1.30	-0.93	-1.04	-1.35	-1.14
**age_2**	0.89	0.61	0.66	0.67	0.70
**age_3**	0.79	0.71	0.84	0.80	0.84
**gender_1**	0.00	-0.25	-0.34	-0.33	-0.29

Note: The Charlson Comorbidity Index (CCI) is calculated using ICD codes from each individual's medical history [1]. The conditions included in the CCI are myocardial infarction, congestive heart failure, cerebrovascular disease, dementia, chronic pulmonary disease, rheumatologic disease, peptic ulcer disease, mild liver disease, diabetes, diabetes with chronic complications, hemiplegia or paraplegia, renal disease, any malignancies, moderate or severe liver disease, metastatic solid tumors, and AIDS. The diagnostic codes and calculation of CCI were performed as described by Deyo et al. [2] and Quan et al. [3]

Additional simulation results:

In order to account for the uncertainties in the comparison of the examined methods, we have conducted several statistical tests to handle the randomness in the simulation results. Recall that the measurements we used to compare methods are the Euclidean distance of the estimate to its true value to see the parameter estimation performance, e.g., the estimation error for the local estimator is calculated as $|| \hat{\beta}_1 - \beta^* ||_2$, and the true positive rate and false positive rate to see the variable selection performance. Since at each replication of the simulation all methods used the same data to generate their parameter estimates, which induces correlation between any two estimates, a paired t-test is appropriate to conduct a pairwise comparison among methods while accounting for this correlation. A paired t-test is commonly used when the two variables under comparison are observed from the same subject which leads to an inherited correlation between them, and the paired t-test takes a direct inspection at their difference by the one-sample t-test to compare the sample mean of the difference to a hypothesized value. In our case, take the comparison between the local estimator and the global estimator as an example, at the ith replication we get an observation of the difference $d_i = d_{i,local} - d_{i,global}$ where $d_{i,local} = || \hat{\beta}_1 - \beta^* ||_2$ and $d_{i,global} = || \hat{\beta}_N - \beta^* ||_2$, and from all 200 replications we have $(d_1, \ldots, d_{200})$. The one-sided one-sample t-test uses the asymptotic relationship between the sample mean $\frac{\sum_{i=1}^{200} d_i}{200}$ and the true mean of d, i.e., $mean(d) = mean(d_{local}) - mean(d_{global})$, to test the following hypothesis

$$H_0: mean(d) \leq 0 \leftrightarrow H_1: mean(d) > 0,$$

which is equivalent to

$$H_0: mean(d_{local}) \leq mean(d_{global}) \leftrightarrow H_1: mean(d_{local}) > mean(d_{global}).$$

We use 0.05 as a significance level and conclude that the local estimator has an inferior estimation performance than the global estimator once the p-value is less than 0.05. As for the comparison of true positive rate and false positive rate, since these two quantities are proportions between 0 and 1, to make their distribution be more normal we applied a logarithmic transformation on them before calculating the differences. Then, a one-sided one-sample t-test is used in the pairwise comparison as in the comparison of the estimation error. This procedure is conducted for selected pairs of methods under all considered settings, and the results are displayed in the following tables.

Supplemental Table 4 The comparison of **estimation error** between pairs of methods and the corresponding test results under **setting 1**.

K	Local-Global	Ave-Global	ADAP1-Global	ADAP2-Global
**5**	0.58*	0.13*	0.05*	0.01*
**10**	0.64*	0.11*	0.06*	0.01*
**20**	0.65*	0.13*	0.06*	0.01*
**30**	0.68*	0.15*	0.07*	0.01*
**40**	0.70*	0.16*	0.07*	0.02*
**50**	0.69*	0.17*	0.08*	0.02*

Note: Each value indicates how much larger the estimation error of the former method is than that of the latter. The symbol "*" denotes a significant one-sided paired t-test result, otherwise there is not enough evidence to reject the null hypothesis. For example, "0.58*" in the first column means that with significance level 0.05 the estimation error of the local estimator is greater than that of the global estimator, and the average difference in estimation error is $\sum_{i=1}^{200} \frac{1}{200}(d_{i,local} - d_{i,global}) = 0.58$. The Supplemental Tables 5 - 7 follow the same formatting.

Supplemental Table 4 (continued)

K	Local-ADAP2	Ave-ADAP2	ADAP1-ADAP2	Local-ADAP1	Ave-ADAP1
**5**	0.57*	0.12*	0.04*	0.53*	0.08*
**10**	0.63*	0.09*	0.05*	0.58*	0.05*
**20**	0.64*	0.12*	0.05*	0.59*	0.07*
**30**	0.66*	0.13*	0.05*	0.61*	0.08*
**40**	0.68*	0.14*	0.05*	0.63*	0.08*
**50**	0.67*	0.15*	0.06*	0.62*	0.09*

Supplemental Table 5 The comparison of **estimation error** between pairs of methods and the corresponding test results under **setting 2**.

K	Local-ADAP2	Ave-ADAP2	ADAP1-ADAP2	Local-ADAP1	Ave-ADAP1
**5**	0.84*	0.10*	0.24*	0.60*	-0.14
**10**	0.81*	0.09*	0.72*	0.09	-0.63
**20**	0.75*	0.11*	0.60*	0.14*	-0.50
**30**	0.95*	0.12*	0.71*	0.24*	-0.59
**40**	0.88*	0.14*	0.84*	0.03	-0.70
**50**	0.84*	0.15*	0.92*	-0.07	-0.77

Supplemental Table 6 The comparison of **estimation error** between pairs of methods and the corresponding test results under **setting 3**.

n_1	Local-ADAP2	Ave-ADAP2	ADAP1-ADAP2	Local-ADAP1	Ave-ADAP1
**500**	1.61*	0.58*	0.11*	1.50*	0.48*
**1000**	0.85*	0.63*	0.03*	0.82*	0.60*
**2000**	0.42*	0.61*	0.00*	0.42*	0.61*
**3000**	0.25*	0.58*	0.00*	0.24*	0.57*
**4000**	0.17*	0.52*	0.00*	0.17*	0.52*
**5000**	0.11*	0.47*	0.00*	0.11*	0.47*
**6000**	0.07*	0.40*	0.00*	0.07*	0.40*
**7000**	0.05*	0.32*	0.00	0.05*	0.32*
**8000**	0.03*	0.24*	-0.00	0.03*	0.24*

Supplemental Table 7 The comparison of **estimation error** between pairs of methods and the corresponding test results under **setting 4.**

n	Local-Global	Ave-Global	ODAL1-Global	ODAL2-Global
**300**	2.03*	1.06*	0.37*	0.12*
**400**	1.73*	0.94*	0.25*	0.06*
**500**	1.53*	0.84*	0.19*	0.05*
**600**	1.34*	0.72*	0.14*	0.05*
**700**	1.22*	0.67*	0.11*	0.03*
**800**	1.12*	0.60*	0.09*	0.03*
**900**	1.01*	0.54*	0.08*	0.03*
**1000**	0.92*	0.50*	0.07*	0.03*
**1100**	0.85*	0.47*	0.06*	0.03*
**1200**	0.80*	0.43*	0.05*	0.02*
**1300**	0.74*	0.41*	0.05*	0.02*

Supplemental Table 7 (continued)

n	Local-ADAP2	Ave-ADAP2	ADAP1-ADAP2	Local-ADAP1	Ave-ADAP1
**300**	1.90*	0.94*	0.25*	1.66*	0.69*
**400**	1.67*	0.88*	0.19*	1.49*	0.69*
**500**	1.47*	0.78*	0.13*	1.34*	0.65*
**600**	1.29*	0.68*	0.10*	1.20*	0.58*
**700**	1.19*	0.64*	0.08*	1.11*	0.55*
**800**	1.08*	0.57*	0.06*	1.03*	0.51*
**900**	0.98*	0.51*	0.05*	0.93*	0.46*
**1000**	0.89*	0.48*	0.04*	0.85*	0.43*
**1100**	0.83*	0.44*	0.03*	0.80*	0.41*
**1200**	0.78*	0.41*	0.03*	0.75*	0.38*
**1300**	0.72*	0.39*	0.03*	0.69*	0.36*

Supplemental Table 8 The comparison of **true positive rate** between pairs of methods and the corresponding test results under **setting 5**.

β	**Ave-Local**	**Global-Local**	**Global-ADAP1**	**Global-ADAP2**	**Ave-Global**
**0.1**	0.65*	0.53*	0.07*	-0.00	0.12*
**0.2**	0.60*	0.60*	0.03*	0.00*	0.00*
**0.3**	0.39*	0.39*	0.00*	0.00	0.00
**0.4**	0.25*	0.25*	0.00	0.00♣	0.00♣
**0.5**	0.16*	0.16*	0.00	0.00♣	0.00♣

Note: Each value shows how much larger the true positive rate of the former method is than that of the latter. The "*" indicates a significant one-sided paired t-test result, otherwise there is not enough evidence to reject the null hypothesis. To avoid singularity, for zero observations a ½ is added. For example, "0.53*" in the second column means that with significance level 0.05 the true positive rate of the global estimator is greater than that of the local estimator and the average difference in true positive rate is 0.53. The symbol "♣" means that the pairwise differences in true positive rate across 400 replications are all zeroes, and the test cannot be conducted. All the following tables follow the same formatting.

Supplemental Table 8 (continued)

β	**ADAP2-Local**	**ADAP2-ADAP1**	**Ave-ADAP1**	**Ave-ADAP2**	**ADAP1-Local**
**0.1**	0.53*	0.07*	0.19*	0.12*	0.45*
**0.2**	0.59*	0.03*	0.04*	0.01*	0.57*
**0.3**	0.39*	0.00*	0.00*	0.00	0.39*
**0.4**	0.25*	0.00	0.00	0.00♣	0.25*
**0.5**	0.16*	0.00	0.00	0.00♣	0.16*

Supplemental Table 9 The comparison of **false positive rate** between pairs of methods and the corresponding test results under **setting 5**.

β	**Ave-Local**	**Global-Local**	**Ave-Global**	**Global-ADAP1**	**ADAP2-Global**
**0.1**	0.40*	0.06*	0.35*	0.01*	0.05*
**0.2**	0.54*	0.04*	0.50*	0.03*	0.04*
**0.3**	0.59*	0.03*	0.55*	0.05*	0.03*
**0.4**	0.61*	0.02*	0.59*	0.05*	0.02*
**0.5**	0.63*	0.03*	0.60*	0.07*	-0.00

Supplemental Table 9 (continued)

β	**ADAP2-Local**	**Ave-ADAP2**	**ADAP2-ADAP1**	**Local-ADAP1**	**Ave-ADAP1**
**0.1**	0.10*	0.30*	0.05*	-0.05	0.35*
**0.2**	0.08*	0.45*	0.07*	-0.01	0.52*
**0.3**	0.07*	0.52*	0.08*	0.01*	0.60*
**0.4**	0.04*	0.57*	0.07*	0.03*	0.64*
**0.5**	0.02*	0.60*	0.07*	0.04*	0.67*

Additional application results:

To account for the uncertainties in the comparison of the examined methods in terms of prediction, we have derived the 95% empirical confidence interval based on AUC values obtained from 200 random-splitting procedures. Specifically, we calculate the difference between the AUC values obtained by ADAP2 and other methods at each random-splitting procedure and then use the empirical 2.5th percentile and 97.5th percentile to construct the 95% confidence interval. Since there could be overlap between the training sets obtained from different splits (same for the testing sets), the paired t-test is not appropriate here. The averaged difference in AUC between pairs of methods accompanied by the corresponding 95% confidence interval is shown below.

Supplemental Table 10 The comparison of **AUC** between pairs of methods and the corresponding 95% confidence interval (CI) in OUD analysis.

Test size	ADAP2-Local	95% CI	ADAP2-Ave	95% CI	ADAP2-ADAP1	95% CI
**1**	0.021	(0.008, 0.034)	0.003	(-0.003, 0.009)	0.004	(-0.002, 0.009)
**2**	0.021	(0.012, 0.031)	0.003	(-0.001, 0.007)	0.004	(-0.000, 0.008)
**3**	0.022	(0.013, 0.032)	0.003	(-0.001, 0.007)	0.004	(0.000, 0.008)
**4**	0.023	(0.014, 0.033)	0.004	(0.000, 0.008)	0.004	(0.000, 0.008)
**5**	0.024	(0.014, 0.038)	0.004	(0.000, 0.008)	0.004	(0.001, 0.009)
**6**	0.026	(0.014, 0.040)	0.004	(0.000, 0.008)	0.004	(0.000, 0.009)
**7**	0.029	(0.012, 0.047)	0.004	(-0.001, 0.011)	0.004	(-0.002, 0.010)
**8**	0.035	(0.016, 0.061)	0.006	(-0.001, 0.013)	0.005	(-0.002, 0.013)
**9**	0.054	(0.022, 0.113)	0.007	(-0.005, 0.020)	0.007	(-0.004, 0.022)

Note: Each value indicates how much larger the AUC of the former method is than that of the latter. For example, "0.021" in the first column means that the average difference in AUC values is 0.021. The numbers in the parentheses denote the empirical 95% confidence interval constructed by the 2.5th percentile and the 97.5th percentile.

References:

1. Charlson ME, Pompei P, Ales KL, et al. A new method of classifying prognostic comorbidity in longitudinal studies: development and validation. *J Chronic Dis.* 1987;40:373–83.
2. Deyo RA, Cherkin DC, Ciol MA. Adapting a clinical comorbidity index for use with ICD-9-CM administrative databases. *J Clin Epidemiol*. 1992;45:613–9.
3. Quan H, Sundararajan V, Halfon P, et al. Coding algorithms for defining Comorbidities in ICD-9-CM and ICD-10 administrative data. *Med Care*. 2005;43(11):1130-9.
